# Do Ruminal Ciliates Select Their Preys and Prokaryotic Symbionts?

**DOI:** 10.3389/fmicb.2018.01710

**Published:** 2018-07-31

**Authors:** Tansol Park, Zhongtang Yu

**Affiliations:** Department of Animal Sciences, The Ohio State University, Columbus, OH, United States

**Keywords:** ruminal ciliates, free-living prokaryotes, ciliate-associated prokaryotes, preys, symbionts

## Abstract

Ruminal ciliates both preys on and form symbiotic relationships with other members of the ruminal microbiota for their survival. However, it remains elusive if they have selectivity over their preys or symbionts. In the present study, we investigated the above selectivity by identifying and comparing the free-living prokaryotes (FLP) and the ciliate-associated prokaryotes (CAP) using Illumina MiSeq sequencing of 16S rRNA gene amplicons. We used single ciliates cells of both monocultures of *Entodinium caudatum* and *Epidinium caudatum* and eight different ciliate genera isolated from fresh rumen fluid of dairy cows. Irrespective of the source (laboratory monocultures vs. fresh isolates) of the single ciliate cells, the CAP significantly differed from the FLP in microbiota community profiles. Many bacterial taxa were either enriched or almost exclusively found in the CAP across most of the ciliate genera. A number of bacteria were also found for the first time as ruminal bacteria in the CAP. However, no clear difference was found in methanogens between the CAP and the FLP, which was confirmed using methanogen-specific qPCR. These results suggest that ruminal ciliates probably select their preys and symbionts, the latter of which has rarely been found among the free-living ruminal prokaryotes. The bacteria enriched or exclusively found in the CAP can be target bacteria to detect and localize using specific probes designed from their 16S rRNA sequences, to characterize using single-cell genomics, or to isolate using new media designed based on genomic information.

## Introduction

Rumen protozoa, exclusively ciliates, rank second only to bacteria in cellular biomass of the ruminal microbiota. They are only found in the rumen and similar habitats (Dehority, 1986, 2005) where they play important roles in feed digestion and homeostasis of the rumen ecosystem (Firkins et al., 2007; Newbold et al., 2015). However, they are also blamed for promoting methane (CH_4_) emission due to their mutualistic relationship with methanogens by producing hydrogen (Newbold et al., 1995). Studies using defaunated (free of ruminal ciliates) sheep and cows also provided strong evidence that ruminal ciliates lower nitrogen utilization efficiency in ruminants by decreasing microbial protein supply to the host small intestine due to constant engulfing of bacteria and subsequent bacterial protein degradation inside the rumen (Fondevila and Dehority, 2001). In the early studies (Morgavi et al., 2006; Mosoni et al., 2011), defaunated animals and faunated animals (carrying normal ruminal ciliates) were shown to have different ruminal microbiota, and the difference was postulated as a result of selective predation on and association with ruminal prokaryotes (Dehority, 2003; Belanche et al., 2011). The predator-prey relationship and the symbiotic relationship between ruminal ciliates and prokaryotes have attracted much research interest because both relationships are of biological interest and potential implication in improving ruminant production.

Studies using fluorescence *in situ* hybridization (FISH) and electron microscopy revealed the presence of methanogens inside and on the surface of ruminal ciliate cells (Vogels et al., 1980; Finlay et al., 1994; Lloyd et al., 1996; Xia et al., 2014). Several studies (Regensbogenova et al., 2004; Tóthová et al., 2008; Tymensen et al., 2012; Belanche et al., 2014) also revealed differences in the methanogens detected inside and outside of ruminal ciliate cells, but no difference was reported in other studies (Chagan et al., 1999; Tokura et al., 1999; Xia et al., 2014). Using cloning and sequencing of 16S rRNA genes, Irbis and Ushida (Irbis and Ushida, 2004) detected *Proteobacteria* associated with single cells of *Polyplastron multivesiculatum*, but the *P. multivesiculatum* cells was pretreated with a mixture of antibiotics, and they did not analyze the free-living bacteria. Studies comparing the bacterial populations between faunated and defaunated sheep revealed differences in bacterial communities, but the difference could not be attributed to selective predation, symbiosis or other microbial interactions, and the bacteria unique to each treatment were not identified (Ozutsumi et al., 2005, 2006; Yánez-Ruiz et al., 2007). Dual symbiosis with both bacteria and methanogens was reported in free-living anaerobic ciliate, *Trimyema compressum*, which belongs to the same subclass *Trichostomatia* (Shinzato et al., 2007) as ruminal ciliates. It remains elusive if ruminal ciliates also form specific association with bacteria and methanogens or select their preys. The lack of axenic cultures of ruminal ciliates and inability to develop or maintain such axenic cultures make it difficult to address the above two questions. Our recent study did show that *Entodinium caudatum*, the most predominant ruminal ciliate species, probably depends on certain prokaryotes for their survival (Park et al., 2017). In the present study, we aimed to identify the prokaryotes that ruminal ciliates select as potential preys or symbionts using monocultures of *Ent. caudatum* and *Epidinium caudatum*, and ruminal ciliates of eight genera (*Dasytricha, Diplodinium, Diploplastron, Entodinium, Epidinium, Isotricha, Ophryoscolex* and *Polyplastron*) isolated from fresh rumen fluid of dairy cows. Our results provided new insight into the relationship between ruminal ciliates and prokaryotes.

## Materials and methods

### Characterization of ciliate-associated vs. free-living prokaryotes in monocultures of ruminal ciliates *Ent. caudatum* and *Epi. caudatum*

The monocultures of *Ent. caudatum* and *Epi. caudatum* used in the present study were initially established from single cells individually picked from the rumen fluid of a gerenuk and a Jersey dairy cow, respectively (Dehority, 2010). Each monoculture has prokaryotes in addition to the single ciliate species. Frozen stock (−80°C in SP medium containing 4% dimethyl sulfoxide) of each monoculture was activated by cultivation in SP medium at 39°C and maintained by transfer twice a week under anaerobic conditions with O_2_-free CO_2_ gas (Dehority, 1998). The ciliate monocultures were fed daily a ciliate feed as a mixture of ground wheat grain, alfalfa and grass hays (Park et al., 2017). The ciliate cells of each monoculture were counted under a microscope 0, 2, 4, and 8 h after feeding and then fixed with 1% formalin (Park et al., 2017). Single cells (1–3) of *Ent. caudatum* and *Epi. caudatum* were individually picked from the respective fixed monocultures using micropipettes under a microscope connected to a Nikon D50 digital camera (Nikon, Inc., Melville, NY). The single cells were serially transferred at least 5 times in a droplet of sterile phosphate-buffered saline (PBS) followed by 3 additional washes in sterile PBS with low degree centrifugation (1,000 × *g* for 3 min) to retain the ciliate cells. The supernatant of the finial wash and centrifugation was subjected to PCR amplification using bacteria- and archaea-specific 16S rRNA gene primers (Lane, 1991) to verify if prokaryotes that were loosely-associated with the ciliate cells on their surface had been completely removed (as indicated by negative PCR amplification). Only those washed ciliate cells that had no amplification of bacterial or methanogen 16S rRNA genes from their washing supernatant were used in the downstream analysis. Total DNA was extracted from the isolated single ciliate cells using Chelex-100 and Proteinase K as described by Irbis and Ushida (2004). Briefly, after the single ciliate cells were digested using Proteinase K at 56°C for 2 h. Following inactivation of the Proteinase K by incubation at 95°C for 10 min, the lysate was centrifuged (21,000 × *g* for 3 min) to separate the supernatant from the cell debris. The supernatant containing the DNA was collected and tested for the presence of ciliate DNA with PCR using ciliate-specific 18S rRNA gene primers (Sylvester et al., 2004) to confirm successful isolation of single ciliate cells. Then, 1 ml of each monoculture was subjected to a series of centrifugation to remove ciliates and prepare FLP. Briefly, 1 ml of each monoculture was centrifuged at 1,000 × *g* for 3 min at room temperature to pellet the ciliate cells while leaving the prokaryotes in the supernatant. The supernatant was transferred to a fresh tube and centrifuged again. The supernatant from the second centrifugation was centrifuged at 16,000 × *g* for 10 min at 4°C to pellet the FLP. Total metagenomic DNA of the FLP was extracted using the repeated bead-beating plus column purification (RBB+C) method as previously described (Yu and Morrison, 2004). DNA extraction was repeated two more times at different days, and the DNA extracts were combined by ciliate species, prokaryotic fractions, and sampling times.

### Identification of prokaryotes associated with single ciliate cells isolated from fresh rumen fluid of dairy cows

Fresh rumen fluid samples were collected 2 h after morning feeding from 5 rumen-cannulated Jersey dairy cows, which were fed a total mixed ration consisting of corn silage, alfalfa hay, and grounded shelled-corn. Formalin was immediately added to the rumen fluid samples to a 1% final concentration to fix the samples. The generic composition of ruminal ciliates in each rumen fluid sample was determined morphologically as described previously (Baraka, 2012; Martinele et al., 2014). For the ease of isolation of single ciliate cells, the formalin-fixed rumen fluid samples were filtered through 50- and 10-μm pore-sized nylon filter membrane sequentially (Sefar Filtration Inc., New York, USA) to separate the small-sized dominant *Entodinium* from other genera. Then, single cells of ciliates were isolated as described above and identified based on their morphologies including cell size, the location of vestibulum and ciliary zone (Baraka, 2012; Martinele et al., 2014). The morphology-based identification was confirmed by sequencing the 18S rRNA gene amplified using ciliate-specific primers (Sylvester et al., 2004) and sequence comparison using BLAST against GenBank. Eight different genera of ruminal ciliates were successfully isolated as single cells and identified to known ruminal ciliate genera. The GenBank accession numbers and sequence identity of the isolated single ruminal ciliate cells, including those of the monocultures of *Ent. caudatum* and *Epi. caudatum*, were shown in Table S1. From the isolated single cells of ciliates and the FLP, metagenomic DNA was extracted as described above.

### Microbiota analysis using amplicon sequencing

The CAP and the FLP were identified using Illumina MiSeq sequencing and analysis of 16S rRNA gene amplicons of the V4–V5 hypervariable regions essentially as described previously (Kigerl et al., 2016). Briefly, primers 515F (5′-GTGCCAGCMGCCGCGGTAA-3′) and 806R (5′-GGACTACHVGGGTWTCTAAT-3′) were used to amplify the V4–V5 region of both bacteria and methanogens (Caporaso et al., 2011). The purified amplicon libraries (each having a unique barcode sequence) of all the DNA extracts were pooled and sequenced using the 2 × 300 paired-end chemistry. The sequence data were processed using QIIME with the default options applied (ver.1.9.1) (Caporaso et al., 2010). The two paired-end reads were joined, and low-quality reads (Q < 25) were filtered out followed by trimming of barcode and primer sequences. Then, the joined sequences shorter than 200 bp or longer than 600 bp were discarded. Sequences with any homopolymers longer than 6 nt were removed, and the quality-checked sequences (Q > 25) were merged into a single Fasta file. Operational taxonomic units were picked using the open-reference OTU picking method against the Greengenes reference set (ver. 13_8) (DeSantis et al., 2006) at 97% similarity using PyNAST (Caporaso et al., 2010). Those sequences that failed to cluster with the Greengenes reference OTUs were clustered into OTUs *de novo* at 97% sequence similarity. Probable chimeric sequences were predicted using ChimeraSlayer with the default options applied against the Greengenes aligned reference sequences (DeSantis et al., 2006) and removed. The OTUs of methanogens were separated from the bacterial OTUs in the Biom OTU tables so that methanogens and bacteria could be analyzed separately. Taxonomic classification was determined using the RDP Classifier with 80% confidence (Wang et al., 2007). Relative abundance of a taxon was expressed as its % of sequences relative to total sequences in respective samples.

α-diversity measurements, including observed number of OTUs, Shannon diversity index (*H*′), Simpson's index of diversity (*1-D*), Chao 1 species richness estimate, and phylogenetic diversity (PD_whole_tree), were calculated from rarefied Biom OTU tables. Principal coordinates analysis (PCoA) based on unweighted UniFrac distances (Lozupone and Knight, 2005) was used to compare the overall microbiota between the CAP and the FLP of all ciliate species. The CAP-specific bacterial 16S rRNA amplicon sequences shared by ciliate single cells were used to generate a maximum likelihood tree using MEGA6 with 1,000 bootstraps (Tamura et al., 2013). The raw sequence reads were deposited into the Sequence Read Archive (SRA) of NCBI under the accession number PRJNA476351.

### Quantification of methanogen abundance using quantitative PCR

The abundance of total methanogens, genus *Methanobrevibacter*, class *Thermoplasmata*, family *Methanosarcinaceae*, and species *Methanosphaera stadtmanae*, all of which were detected in the CAP and the FLP of the isolated ciliate single cells, was quantified as copies of 16S rRNA genes using respective group-specific primer sets and a nested PCR approach as described by Tymensen et al. (2012). A nested PCR was used because of the low abundance of methanogens in the CAP extracts. Briefly, amplicons representing total methanogens were generated from each of the DNA extracts using PCR and primers Met86f/Met915r (Wright and Pimm, 2003; Watanabe et al., 2004) and then purified using a QIAquick PCR purification kit (Qiagen, Inc., Valencia, CA). The purified PCR product (diluted 1:100) was used as the enriched methanogens 16S rRNA gene templates for qPCR quantification. One sample-derived qPCR standard (Yu et al., 2005) was prepared for each methanogen group using PCR with respective group-specific primers (Table S2) using a DNA extract from one FLP sample that had all the methanogen targets detected in the sequencing analysis. Ten-fold serial dilutions (10^1^-10^9^ 16S rRNA gene amplicons per μl) were prepared for each qPCR standard and used in qPCR analysis as described previously (Wang et al., 2012). The qPCR cycles included initial denaturation at 95°C for 10 min, followed by 40 cycles (95°C for 30 s, 60°C for 30 s, 72°C for 30 s) and a final extension for 5 min at 72°C. For enumeration of *Methanobrevibacter* spp., the annealing temperature was 63°C. Melting curves generated between 52 and 95°C in each run were checked to verify the specificity of qPCR amplification.

### Statistical analysis

For the monocultures of *Ent. caudatum* and *Epi. caudatum*, the relative abundance of each taxon and the abundance of the quantified methanogen groups in the two prokaryotic fractions (FLP vs. CAP) at different time after feeding (0, 2, 4, and 8 h) were statistically analyzed using the GLIMMIX procedure of SAS 9.4 (SAS Institute Inc., Cary, NC). Prokaryotic fraction x time post feeding (fraction × time) interaction was also examined. For the single ciliate cells from the two ciliate monocultures, a polynomial contrast was used to analyze the population shift of the CAP and the FLP over time of incubation. Relative abundance of taxa among ciliate genera was compared using the GLIMMIX procedure of SAS with animal as a random effect. The Scheffe *post-hoc* test was used to examine significant differences between the FLP and CAP from the rumen fluid. For the monocultures of *Ent. caudatum* and *Epi. caudatum* and the fresh rumen fluid, significant differences in the overall microbiota between the CAP and the FLP were estimated using ANOSIM (Clarke and Gorley, 2006) implemented in QIIME based on the unweighted UniFrac distances. The significance was declared at *P* ≤ 0.05.

## Results

### Free-living and ciliate-associated prokaryotes in the monocultures of *Ent. caudatum* and *Epi. caudatum*

#### Bacteria

**Figure 1:**
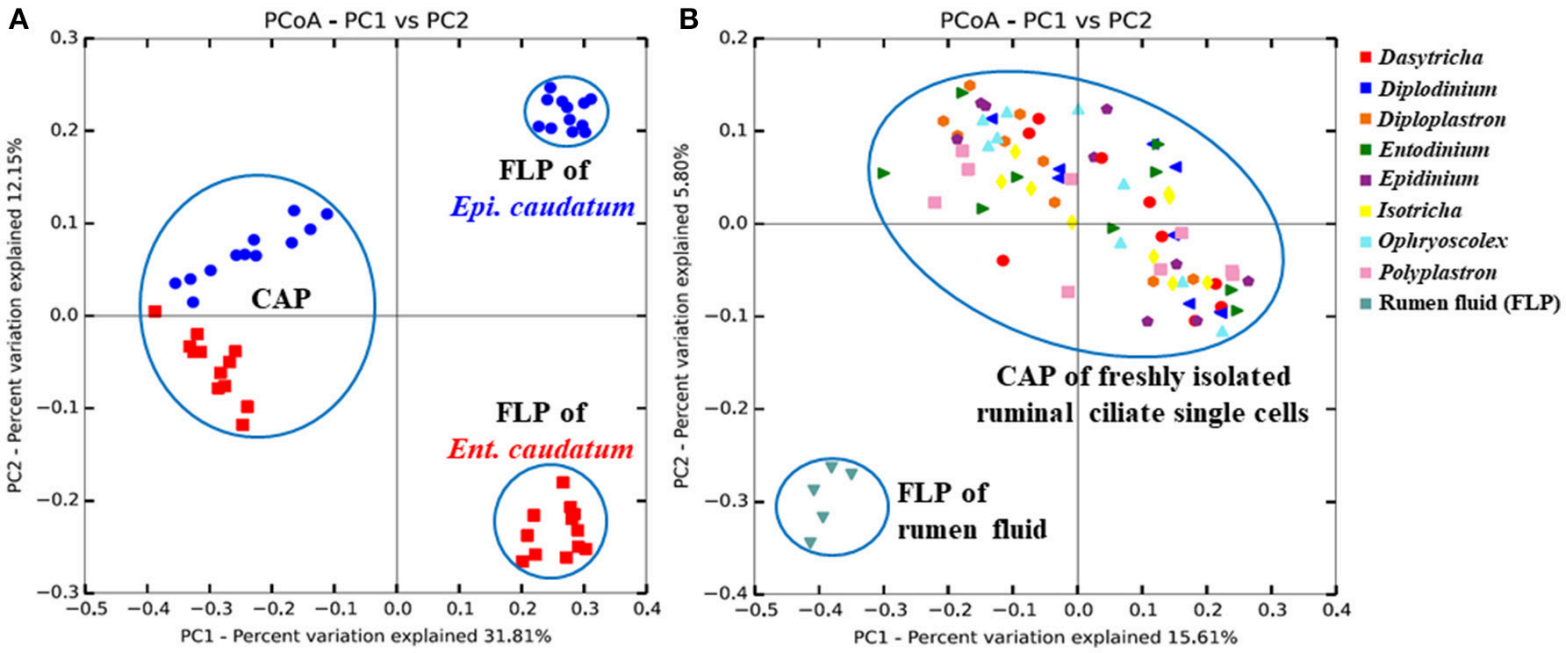
Principal coordinates analysis based on unweighted UniFrac distances in **(A)** both the FLP and CAP of two ciliate monocultures [*Ent. caudatum* (red squares) and *Epi. caudatum* (blue spheres)] and **(B)** the CAP of fresh isolates of ruminal ciliate singles cells and the FLP of the rumen fluid collected from Jersey dairy cows. Based on ANOSIM, FLP differed (*P* < 0.001) from CAP of both the monocultures and the rumen fluid.

**Figure 2:**
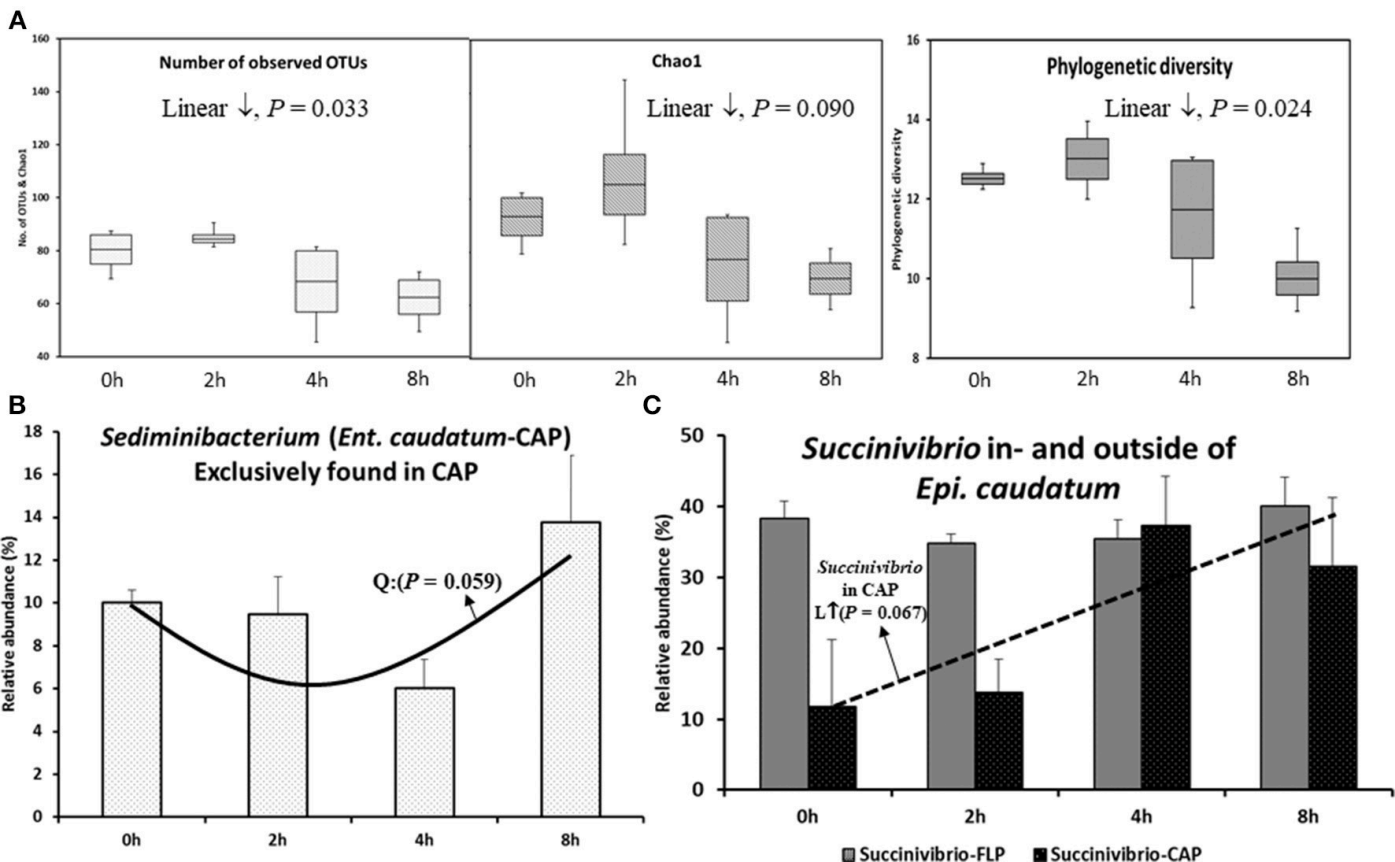
Temporal changes in species richness, phylogenetic diversity, and relative abundance of select taxa after feeding. **(A)** α-diversity measurements in the CAP of *Ent. caudatum*. **(B)** *Sediminibacterium* in the CAP of *Ent. caudatum*, and **(C)** *Succinivibrio* found in- and outside of *Epi. caudatum* cells. No significant temporal changes of α-diversity measurements were found in *Epi. caudatum*.

Except for the Shannon-Wiener diversity index of both ciliate monocultures and the Simpson diversity index of the *Epi. caudatum* monoculture, all the α-diversity measurements differed between the CAP and the FLP (Table 1). More operational taxonomic units (OTUs) were found and predicted (Chao1 richness) in the FLP than in the CAP even though the FLP had a lower depth coverage than the CAP. On the PCoA plot, the CAP and the FLP of each ciliate monoculture were separated along PC1, while the CAP and the FLP of both ciliate monocultures were separated along PC2 (Figure 1A). Based on the analysis of similarity (ANOSIM) method, the CAP differed (*P* < 0.001) from the FLP for both ciliate monocultures, and the two ciliate monocultures had different (*P* < 0.001) prokaryotic populations for both the CAP and the FLP. No significant change was found in all the α-diversity measurements of the CAP for both ciliate monocultures over the 8 h after feeding, except a linear decrease in the number of observed OTUs (*P* = 0.033), a tendency of linear decrease in Chao1 species richness estimate (*P* = 0.090), and phylogenetic diversity (*P* = 0.024) of the *Ent. caudatum* monoculture (Figure 2A). In the FLP of both monocultures, no significant postprandial change in both relative abundance of major taxa (>0.5% of total sequences) and all the α-diversity measurements (data not shown).

**Table 1:** Summary of α-diversity measurements.

**Ciliate genera**	Prokaryotic fraction^*^	**Raw sequences**	**Quality sequences**	**No. of OTUs^#^**	**Chao1**	**Simpson**	**Shannon**	**Phylogenetic diversity**	**Goods Coverage (%)**
**MONOCULTURES**									
*Entodinium caudatum*	FLP	80,257^a^	5,616^a^	125^a^	248^a^	0.91^b^	5.09	16.6^a^	86.1^b^
	CAP	14,882^b^	1,182^b^	78^b^	96^b^	0.95^a^	5.24	12.3^b^	95.9^a^
*Epidinium caudatum*	FLP	66,454^a^	6,505^a^	102^a^	230^a^	0.85	4.35	15.4^a^	88.1^b^
	CAP	15,241^b^	1,906^b^	75^b^	92^b^	0.89	4.71	12.8^b^	95.7^a^
**FRESHLY ISOLATED CILIATES**									
Rumen fluid	FLP	43,316	33,630	269^a^	443^a^	0.95	6.46^a^	26.3	90.1^b^
*Dasytricha*	CAP	47,162	15,239	80^b^	96^b^	0.96	5.33^b^	13.5^c^	98.9^a^
*Diplodinium*	CAP	28,224	10,505	87^b^	100^b^	0.97	5.45^b^	14.3^bc^	98.8^a^
*Diploplastron*	CAP	46,066	24,845	130^b^	144^b^	0.97	6.02^ab^	19.6^b^	98.3^a^
*Entodinium*	CAP	39,364	20,029	111^b^	128^b^	0.96	5.56^b^	16.2^bc^	98.2^a^
*Epidinium*	CAP	32,375	16,601	95^b^	110^b^	0.97	5.66^b^	15.4^bc^	98.8^a^
*Isotricha*	CAP	34,608	12,699	97^b^	111^b^	0.96	5.56^b^	15.5^bc^	98.7^a^
*Ophryoscolex*	CAP	35,344	16,677	109^b^	127^b^	0.97	5.68^b^	17.4^bc^	98.3^a^
*Polyplastron*	CAP	44,884	21,163	99^b^	116^b^	0.94	5.39^b^	15.7^bc^	98.5^a^

* *FLP, free-living prokaryotes; CAP, ciliate-associated prokaryotes. Means with different superscripts within columns between the FLP and the CAP of each monoculture or between the FLP and the CAP of the fresh ciliate isolates differ (P < 0.05)*.

# *Singleton was removed*.

In both the CAP and the FLP of the two ciliate monocultures, the relative abundance of all the identified taxa at all the taxonomic ranks did not change over the 8 h after feeding, except that the relative abundance of *Sediminibacterium*, a genus of *Bacteroidetes*, in the CAP of *Ent. caudatum* monoculture quadratically decreased (*P* = 0.059) (Figure 2B), and that of *Succinivibrio* tended (*P* = 0.067) to linearly increase in the CAP of the *Epi. caudatum* monoculture (Figure 2C).

**Figure 3:**
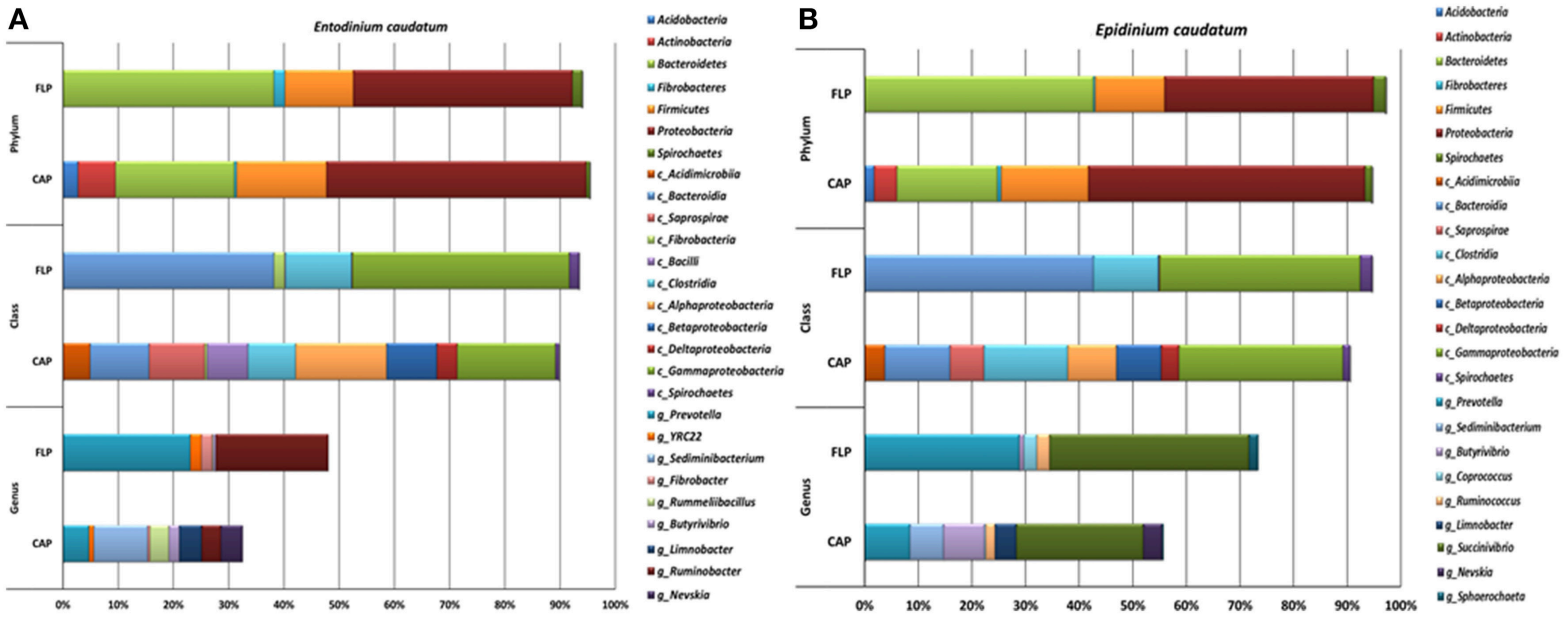
Relative abundance of bacterial phyla, classes and known genera in the CAP and the FLP of the monocultures of *Ent. caudatum* **(A)** and *Epi. caudatum* **(B)** Relative abundance did not add up to 100% because unclassified bacterial taxa were not included.

The bacterial profiles differed to a greater extent at low (e.g., genus) than at high taxonomic ranks (e.g., phylum) between the two fractions and between the two ciliate monocultures (Figure 3 and Table 2). Among the detected bacterial phyla, *Acidobacteria, Actinobacteria, Cyanobacteria, Planctomycetes*, and *Proteobacteria* were significantly more predominant (*P* < 0.05) in the CAP than in the FLP of both ciliate monocultures, whereas *Bacteroidetes* and *Spirochaetes* showed the opposite distributions. *Bacteroidetes* and *Proteobacteria* were the two largest phyla in both the CAP and the FLP, and they exhibited opposite distributions between the two prokaryotic fractions. *Fibrobacteres* had a greater relative abundance in the FLP than in the CAP of the *Ent. caudatum* monoculture but similar predominance between the two prokaryotic fractions of the *Epi. caudatum* monoculture. *Bacteroidia* was about three times more predominant in the FLP than in the CAP (Figure 3). Among *Proteobacteria*, the classes α-, β-, and δ*-Proteobacteria* were nearly exclusively found in the CAP, whereas γ*-Proteobacteria* was found in both the CAP and the FLP of both ciliate monocultures, but it was much more predominant in the FLP. Among the known genera detected, *Prevotella* and *Ruminobacter* were the first and the second largest genera in the *Ent. caudatum* monoculture, while *Succinivibrio* and *Prevotella* were the first and the second largest genera in the *Epi. caudatum* monoculture. For both ciliate monocultures, *Prevotella* was more predominant in the FLP than in the CAP, whereas the opposite was true for *Sediminibacterium, Butyrivibrio, Limnobacter, Perlucidibaca*, and *Nevskia. Coprococcus* displayed opposite distribution in the CAP and the FLP between the two ciliate monocultures, being more predominant in the CAP of *Ent. caudatum monoculture* but more predominant in the FLP of *Epi. caudatum* monoculture. *Fibrobacter* was the sole known genus in *Fibrobacteres* detected, and it was significantly more predominant in the FLP than in the CAP of the *Ent. caudatum* monoculture. No significant temporal differences in relative abundance of the major bacterial taxa (>1% of total sequences) were found for both fractions and both ciliate monocultures (data not shown).

**Table 2:** Relative abundances (%) of major known bacterial taxa^*^ in the FLP and the CAP of the monocultures of *Ent. caudatum* and *Epi. caudatum*.

	Relative abundance (%)^*^				***P*****-value**			
**Phylum**								
Lowest taxa assigned^**^	***Ent. caudatum***		***Epi. caudatum***		***Ent*. vs. *Epi***.	**CAP vs. FLP**	**Species × fraction**	**Detection in CAP^#^**
	**FLP**	**CAP**	**FLP**	**CAP**				
*Acidobacteria*	0	2.43	0	1.79	0.251	<0.001	0.251	Only
o_*Ellin6513*	0	1.40	0	0.71	0.058	<0.001	0.058	Only
*Actinobacteria*	0.02	6.89	0	4.38	0.051	<0.001	0.054	Enriched
c_*Actinobacteria*	0	1.75	0	0.42	0.101	0.009	0.101	Only
o_*Acidimicrobiales*	0	5.10	0	3.96	0.260	<0.001	0.260	Only
*Bacteroidetes*	38.6	21.0	44.0	18.8	0.312	<0.001	0.020	
o_*Bacteroidales*	38.6	10.2	44.0	12.1	0.021	<0.001	0.241	
f_*BS11*	5.71	0.42	2.35	0.03	<0.001	<0.001	<0.001	
f_*Paraprevotellaceae*	2.01	1.03	0.31	0.34	<0.001	0.057	0.042	
g_*Prevotella*	23.5	4.78	29.8	8.54	0.015	<0.001	0.529	
g_*Sediminibacterium*	0	9.99	0	6.47	0.028	<0.001	0.028	Only
*Cyanobacteria*	0	0.80	0.02	1.82	0.118	<0.001	0.130	Enriched
*Fibrobacteres*	1.64	0.43	0.31	0.37	0.003	0.014	0.007	
*Firmicutes*	12.1	15.5	12.7	15.4	0.901	0.130	0.876	
c_*Bacilli*	0.15	6.93	0.13	0.31	0.020	0.015	0.020	Enriched
o_*Bacillales*	0.15	5.49	0	0.21	0.045	0.041	0.058	Enriched
o_*Clostridiales*	11.7	8.38	11.7	14.8	0.024	0.930	0.024	
f_*Planococcaceae*	0.15	3.68	0	0.03	0.143	0.168	0.175	
f_*Lachnospiraceae*	6.18	4.05	3.54	8.61	0.366	0.167	0.001	
g_*Butyrivibrio*	0.48	1.91	0.82	7.24	0.004	<0.001	0.009	Enriched
g_*Coprococcus*	0	0.52	2.16	0.35	<0.001	0.021	<0.001	
f_*Ruminococcaceae*	1.88	1.61	3.62	2.59	0.005	0.168	0.420	
g_*Ruminococcus*	0.29	0.93	2.46	1.79	<0.001	0.964	0.070	
*Planctomycetes*^†^	0.06	1.77	0.10	1.54	0.622	<0.001	0.511	Enriched
c_*Planctomycetia*	0.06	1.08	0.10	1.16	0.799	<0.001	0.913	Enriched
f_*Isosphaeraceae*	0	1.03	0	1.08	0.911	<0.001	0.911	Only
*Proteobacteria*	40.1	48.7	38.4	53.0	0.561	<0.001	0.193	Enriched
c_*α-Proteobacteria*	0	16.3	0	9.23	0.020	<0.001	0.020	Only
o_*Ellin329*	0	5.66	0	3.51	0.124	<0.001	0.124	Only
o_*Rhizobiales*	0	6.03	0	3.36	0.058	<0.001	0.058	Only
f_*Bradyrhizobiaceae*	0	4.60	0	2.37	0.026	<0.001	0.026	Only
f_*Rhodospirillaceae*	0	3.17	0	1.66	0.012	<0.001	0.012	Only
c_*β-Proteobacteria*	0.15	9.57	0.06	7.90	0.372	<0.001	0.417	Enriched
o_*Burkholderiales*	0.15	6.15	0.06	5.34	0.507	<0.001	0.587	Enriched
f_*Comamonadaceae*	0.05	5.94	0	5.04	0.479	<0.001	0.525	Enriched
g_*Limnobacter*	0	3.94	0	3.68	0.857	<0.001	0.857	Only
c_*δ-Proteobacteria*	0.06	4.20	0.24	3.60	0.659	<0.001	0.417	Enriched
o_*Myxococcales*	0	4.07	0	3.04	0.234	<0.001	0.234	Only
f_*0319-6G20*	0	2.57	0	1.63	0.112	<0.001	0.112	Only
c_*γ-Proteobacteria*	39.7	18.6	36.6	32.0	0.035	<0.001	0.001	
f_*Succinivibrionaceae*^†^	39.6	7.35	36.4	24.6	0.013	<0.001	<0.001	
g_*Ruminobacter*	20.7	3.96	0	0.10	<0.001	<0.001	<0.001	
g_*Succinivibrio*	0.27	0.35	36.3	24.4	<0.001	0.020	0.019	
f_*Moraxellaceae*^†^	0.02	1.71	0	1.08	0.129	<0.001	0.148	Enriched
g_*Perlucidibaca*	0	1.30	0	0.74	0.191	<0.001	0.191	Only
o_*Xanthomonadales*	0	9.22	0	6.08	0.072	<0.001	0.072	Only
f_*Sinobacteraceae*	0	7.88	0	5.66	0.101	<0.001	0.101	Only
g_*Nevskia*	0	4.04	0	3.76	0.789	<0.001	0.789	Only
*Spirochaetes*	1.69	0.66	2.04	1.14	0.316	0.024	0.877	
g_*Sphaerochaeta*	1.29	0.08	1.51	0.21	0.595	<0.001	0.885	

* *Only the taxa with a relative abundance greater than 1% at the lowest taxonomic rank were shown*.

** *c, Class; o, Order; f, Family; g, Genus*.

# *Found only or significantly enriched in the CAP in both monocultures*.

† *For those taxa, only the lowest classifiable taxa were shown because their higher taxa had similar relative abundance as the taxa shown*.

**Figure 4:**
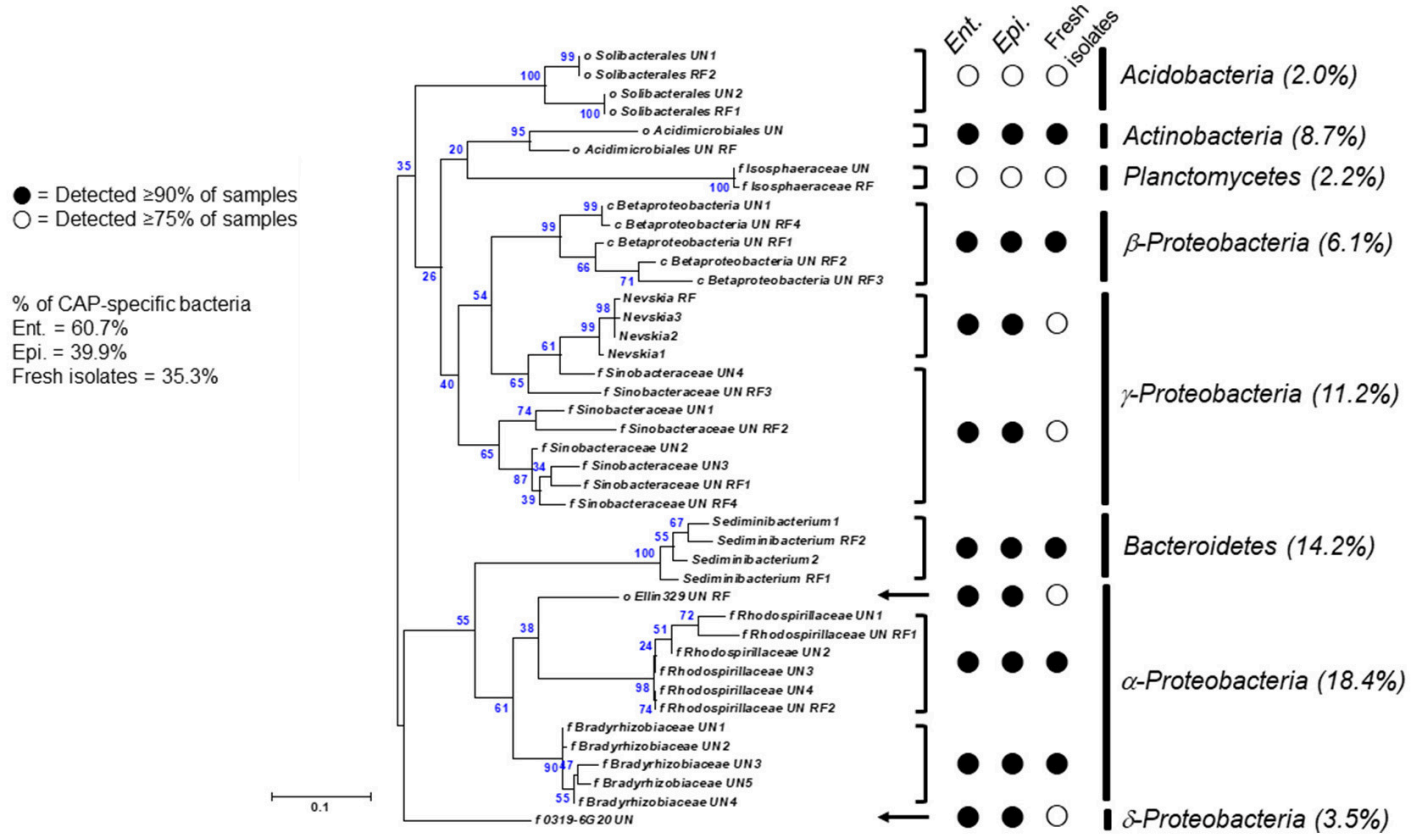
A maximum-likelihood tree showing CAP-specific bacterial 16S rRNA gene sequences shared by ciliate single cells [18 sequences from the freshly isolated ciliate single cells and 24 sequences from *Ent. caudatum* (*Ent*.) and *Epi. caudatum* (*Epi*.) in their monocultures]. Relative abundance of each taxon among CAP-specific bacteria was shown in parentheses. Minor taxa (< 0.5% of total sequences) were excluded.

The *Ent. caudatum* and *Epi. caudatum* single cells shared nine taxa that were detected only in the CAP (Figure 4). Two phyla, *Acidobacteria* and *Actinobacteria*, were almost exclusively found in the CAP of both ciliate species. Among the *Bacteroidetes*, the genus *Sediminibacterium* was only found in the CAP. One candidate order (*Ellin*329), 2 families (*Bradyrhizobiaceae* and *Rhodospirillaceae*) of α*-Proteobacteria*, and one candidate family of γ*-Proteobacteria* (*Sinobacteraceae* UN1) were CAP-specific taxa. Of all the CAP, 60.7 and 39.9% were exclusively found within the CAP of *Ent. caudatum* and *Epi. caudatum* cells, respectively. The two ciliate monocultures also had a different relative abundance of several genera, including *Prevotella, Sediminibacterium, Butyrivibrio, Coprococcus, Ruminococcus, Ruminobacter*, and *Succinivibrio* (Table 2).

#### Methanogens

Methanogens were represented by < 1% of the total sequences for both ciliate monocultures (*Ent. caudatum* = 0.54%, *Epi. caudatum* = 0.73%), all of which were assigned to the phylum *Euryarchaeota* (Table 3A). *Methanobrevibacter* and class *Thermoplasmata* were the largest taxa found in both ciliate monocultures without significant temporal variation in abundances after feeding, and these two taxa had >3-fold greater predominance in the FLP than in the CAP of the *Ent. caudatum* monoculture but similar relative abundance in the two prokaryotic fractions of *Epi. caudatum* monoculture. Among the 12 isolated single cells of each ciliate species, seven of the *Ent. caudatum* cells and 4 of the *Epi. caudatum* cells yielded no methanogen sequences. In both ciliate monocultures, *Methanosphaera* was only found in the FLP at low frequency.

Table 3Relative abundance (%) of methanogen taxa in the FLP and the CAP of the monocultures of *Ent. caudatum* and *Epi. caudatum* **(A)** and the fresh isolates of ruminal ciliates (B)^*^.

**Table:** 

**(A)**												
	***Ent. caudatum***						***Epi. caudatum***					
**Taxa**	**FLP**	**CAP**	**SEM**	**Fraction**	**Time**	F^*^T	**FLP**	**CAP**	**SEM**	**Fraction**	**Time**	F^*^T
*Methanobrevibacter*	0.51	0.14	0.07	0.01	0.08	0.21	0.47	0.26	0.11	0.42	0.95	0.89
*Methanosphaera*	0.03	0	0.01	0.18	0.58	0.58	0.03	0	0.01	0.18	0.58	0.58
*Thermoplasmata*	0.31	0.08	0.07	0.12	0.61	0.71	0.35	0.35	0.08	1.00	0.52	0.61

**Table:** 

**(B)**											
	**CAP of individual ruminal ciliate genera**								**FLP**	**SEM**	**No. of samples detected**
**Taxa**	***Dasytricha***	***Diplodinium***	***Diploplastron***	***Entodinium***	***Epidinium***	***Isotricha***	***Ophryoscolex***	***Polyplastron***			
*Methanobrevibacter*	0.12	0.04	0.57	0.22	0.73	0.21	0.56	0.10	0.30	0.07	25/72
*Methanimicrococcus*	0	0	0.56	0	0	0	0	0	0	0.07	1/72
*Thermoplasmata*	0.01	0	0.09	0.14	0.10	0.10	0	0.26	0.11	0.04	8/72
Overall	0.13	0.04	1.22	0.36	0.83	0.31	0.56	0.37	0.41	0.10	32/72

* *Data are shown as mean of relative abundance (%); n = 3 each for the FLP and the CAP of monocultures; n = 9 for the CAP of the fresh ruminal ciliate isolates; and n = 5 for the FLP of the rumen fluid sample*.

The abundance of total methanogens and four methanogenic archaeal taxa (*Methanobrevibacter, Thermoplasmata, Methanosarcinaceae*, and *Methanosphaera stadtmanae*) were quantified using specific qPCR. The relative abundance of *Methanosarcinaceae* was less than 0.001% of total methanogens, and it was not further discussed. The relative (relative to total prokaryotes) abundance of total methanogens determined by qPCR was in line with that determined from the sequencing data (Table 4). Compared with the CAP, the FLP of the *Epi. caudatum* monoculture had a greater relative abundance of total methanogens, *Thermoplasmata*, and *M. stadtmanae* (Table 4). The two prokaryotic fractions of *Ent. caudatum* monoculture had a similar relative abundance of all the groups of methanogens quantified. The relative abundance of *Thermoplasmata* in the CAP of the *Ent. caudatum* monoculture cubically fluctuated temporally after feeding, while that of *Methanobrevibacter* of the *Epi. caudatum* monoculture decreased overtime linearly after feeding (Figure S1).

**Table 4:** Relative abundance (%) of methanogenic taxa in the monocultures of *Ent. caudatum* and *Epi. caudatum* as quantified using qPCR.

	**FLP**				**CAP**				**SEM**	***P*****-value**		
	**0 h**	**2 h**	**4 h**	**8 h**	**0 h**	**2 h**	**4 h**	**8 h**		**Fraction**	**Time**	F^*^T
***Ent. caudatum***												
Total archaea	0.283	0.326	0.346	0.303	0.203	0.194	0.396	0.144	0.028	0.151	0.251	0.536
*Methanobrevibacter*	0.110	0.118	0.140	0.141	0.191	0.210	0.304	<0.001	0.026	0.323	0.209	0.175
*Thermoplasmata*	0.065	0.111	0.116	0.105	<0.001	0.126	0.018	0.083	0.015	0.166	0.239	0.563
*M. stadtmanae*^#^	0.069	0.117	0.113	0.055	0.074	0.096	0	0	0.016	0.176	0.405	0.612
***Epi. caudatum***												
Total archaea	0.314	0.410	0.352	0.262	0.191	0.093	0.084	0.066	0.041	0.008	0.819	0.812
*Methanobrevibacter*	0.131	0.117	0.118	0.077	0.196	0.021	0.023	0.003	0.019	0.163	0.093	0.307
*Thermoplasmata*	0.108	0.155	0.105	0.068	0.011	0.052	0.074	0.056	0.012	0.010	0.386	0.326
*M. stadtmanae*^#^	0.146	0.256	0.056	0.050	<0.001	0	<0.001	0	0.032	0.061	0.630	0.630

* *Data are shown as means (n = 3) of relative abundance of individual taxa relative to total prokaryotes*.

# *Methanosphaera stadtmanae*.

### Free-living and ciliate-associated prokaryotes in the fresh rumen fluid

#### Ciliate populations

We found and identified eight genera of ruminal ciliates from the rumen fluid collected from dairy cows fed a typical lactating diet (Figure S2). Morphological identification of these genera was confirmed by sequencing their 18S rRNA genes. *Entodinium* dominated the total ciliates in all the five cows sampled, accounting for greater than 94% of the total ciliates (Table S3).

#### Bacteria

More than 1.2 million quality-checked sequences afforded a depth coverage >98% of the CAP of the single ciliate cells isolated from fresh rumen fluid (referred to as single ciliate cells of fresh rumen fluid henceforth) (Table 1). Overall, these single cells had smaller α-diversity measurements of prokaryotes than the rumen fluid except for Simpson's diversity index. On the PCoA plot (Figure 1B), the FLP of rumen fluid formed a cluster that is separate from that of the CAP of the fresh rumen fluid along both PC1 and PC2. Based on ANOSIM, the overall FLP and the CAP of rumen fluid were distinct (*P* < 0.001).

**Figure 5:**
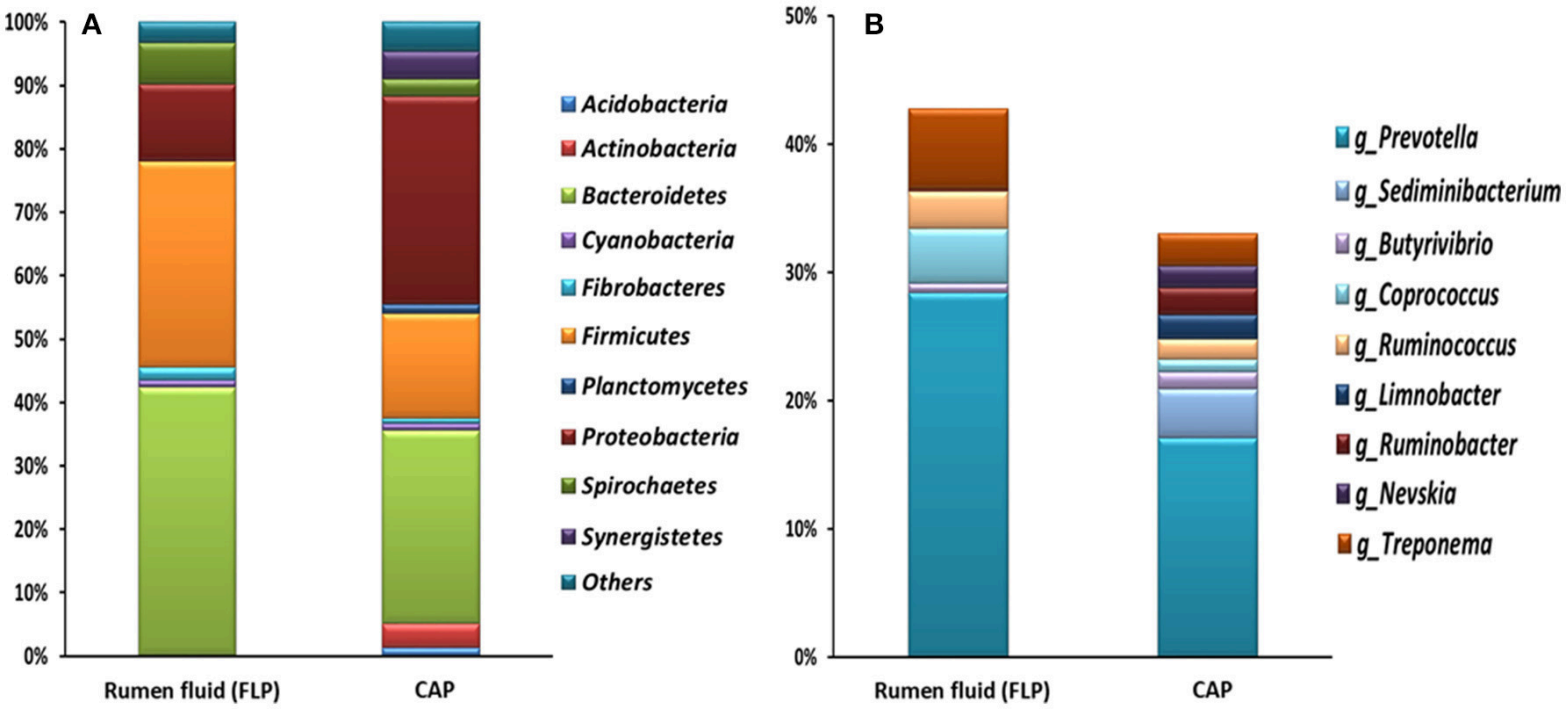
Relative abundance of major bacterial phyla **(A)** and genera **(B)** (each representing >1% of total sequences) of the CAP of freshly isolated ruminal ciliate single cells (average of 8 genera) and the FLP of the rumen fluid collected from Jersey dairy cows. Relative abundance of the genera did not add up to 100% because unclassified bacterial taxa were not included.

Overall, the CAP had a greater predominance of *Proteobacteria* than the FLP, whereas the latter had a greater predominance of *Bacteroidetes, Firmicutes*, and *Fibrobacteres* than the former (Figure 5A). Compared to the FLP, the CAP also had a greater relative abundance of *Acidobacteria* (1.38 vs. 0%, *P* = 0.048), *Actinobacteria* (3.74 vs. 0.08%, *P* = 0.021) and *Proteobacteria* (32.8 vs. 12.2%, *P* < 0.001), but lower relative abundance of *Bacteroidetes* (30.5 vs. 42.4%, *P* = 0.003), *Firmicutes* (16.6 vs. 32.4%, *P* < 0.001) and *Spirochaetes* (2.69 vs. 6.45%, *P* = 0.005). Among the known genera detected, *Prevotella* (28.4 vs. 17.1%, *P* < 0.001) and *Coprococcus* (4.26 vs. 0.97%, *P* = 0.054) were more predominant in the FLP than in the CAP, whereas *Sediminibacterium, Limnobacter*, and *Nevskia* were only detected in the CAP (Figure 5B and Table 5). However, despite the overall difference between the FLP and the CAP, the distribution of each assigned taxon varied depending on the genera of the host ciliates (Table 5). Thirteen major bacterial taxa (each represented by >0.5% of total sequences) were exclusively found in the CAP of the fresh rumen fluid, and 11 of those taxa were also found to be CAP-specific in the *Ent. caudatum* and the *Epi. caudatum* monocultures (Figure 4). These “common” CAP-specific bacterial sequences were found in at least 75% of the replicated CAP samples and were not detected at all in the FLP. *Sediminibacterium*, two different families of α*-Proteobacteria*, unclassified β*-Proteobacteria*, and unclassified *Acidimicrobiales* were shared by 90% of all CAP samples of both monocultures of *Ent. caudatum* and *Epi. caudatum* and the CAP of the fresh rumen fluid.

**Table 5:** Relative abundances (%) of major known bacterial taxa^*^ in the FLP and the CAP of freshly isolated ruminal ciliates.

**Phylum**												
Lowest taxa assigned^**^	**CAP of individual ciliate genera**								**FLP**	***P-*****value**		
	***Dasytricha***	***Diplodinium***	***Diploplastron***	***Entodinium***	***Epidinium***	***Isotricha***	***Ophryoscolex***	***Polyplastron***		**Genera**	**CAP vs. FLP**	**Detection in CAP^#^**
*Acidobacteria*	1.72	1.86	1.48	1.38	1.03	0.92	0.98	1.68	0	NS	0.048	Only
*Actinobacteria*	4.71	4.09	3.77	3.25	2.32	3.59	5.79	2.37	0.08	NS	0.021	Enriched
o_*Acidimicrobiales*	3.41	3.58	3.49	3.16	1.98	2.84	4.12	1.66	0	NS	NS	Only
*Bacteroidetes*	30.9	28.6	35.2	29.1	33.4	31.2	30.2	25.5	42.4	NS	0.003	
c_*Bacteroidia*	25.9	23.8	30.2	24.8	29.2	26.1	24.3	23.1	42.4	NS	0.048	
f_*BS11*	2.03	1.15	1.47	2.19	1.43	1.19	0.81	1.43	0.05	NS	0.040	Enriched
g_*Prevotella*^†^	15.9	14.3	21.5	15.8	19.7	18.6	16.6	14.4	28.4	NS	<0.001	
f_*Paraprevotellaceae*	0.76	0.92	1.62	0.89	1.33	1.52	1.55	1.68	4.96	NS	NS	
f_*Chitinophagaceae*	4.90	3.94	4.04	4.25	4.05	4.84	5.10	2.15	0	0.057	NS	Only
g_*Sediminibacterium*	4.78	3.63	3.62	3.53	3.54	4.22	4.70	2.14	0	NS	0.032	Only
*Cyanobacteria*	2.81	0.82	0.59	1.37	0.37	0.68	1.29	0.50	1.09	NS	NS	
*Fibrobacteres*	1.93	0.32	0.78	0.34	1.01	0.48	1.21	0.31	2.08	NS	NS	
*Firmicutes*	16.2	17.9	15.9	13.1	18.5	19.8	15.0	16.3	32.4	NS	<0.001	
o_*Clostridiales*	14.9	16.6	14.9	12.0	15.9	19.2	13.3	15.2	30.5	0.071	<0.001	
f_*Lachnospiraceae*	8.57	8.51	6.79	6.87	7.75	14.8	7.12	6.71	17.4	NS	0.032	
g_*Butyrivibrio*	1.89	1.54	0.73	1.59	1.58	1.32	1.62	0.38	0.74	0.011	<0.001	Enriched
g_*Coprococcus*	0.77	0.94	0.93	1.28	1.44	0.38	0.90	1.15	4.26	NS	0.054	
f_*Ruminococcaceae*	2.73	3.41	3.78	1.82	3.23	1.69	2.29	3.62	4.82	NS	<0.001	
g_*Ruminococcus*	1.80	1.12	2.59	1.36	1.85	0.98	1.07	2.20	2.93	NS	NS	
f_*Veillonellaceae*	1.63	2.01	1.01	0.57	1.74	1.42	1.35	1.98	0.85	NS	0.095	
*Planctomycetes*	1.57	2.07	2.20	1.16	1.59	0.87	1.11	1.30	0.02	NS	NS	
*Proteobacteria*	31.4	35.9	24.2	34.3	33.2	29.8	29.9	43.9	12.2	0.071	<0.001	Enriched
c_*α-Proteobacteria*	7.57	7.80	4.52	6.14	7.75	5.41	5.96	13.1	0.63	NS	0.021	
o_*Ellin329*	2.34	1.57	1.00	1.84	2.86	1.20	1.71	2.20	0	0.035	0.005	Only
o_*Rhizobiales*	1.36	2.23	1.37	1.06	1.36	1.65	1.74	1.71	0	NS	NS	Only
f_*Bradyrhizobiaceae*	1.09	1.43	0.91	0.85	0.69	1.46	1.29	1.45	0	NS	0.036	*Only*
f_*Rhodospirillaceae*^†^	2.88	3.60	1.26	1.90	2.27	2.08	1.88	1.60	0	NS	0.020	Only
c_*β-Proteobacteria*	5.22	6.24	5.37	3.80	5.04	6.90	7.27	5.39	0.08	NS	0.003	Enriched
o_*Burkholderiales*	2.66	3.85	2.83	2.16	2.80	4.66	2.91	4.09	0.08	NS	<0.001	Enriched
g_*Limnobacter*	1.51	2.51	2.06	1.01	1.77	2.84	1.68	1.71	0	NS	0.054	Only
c_*δ-Proteobacteria*	1.66	2.83	2.91	2.51	2.40	3.09	2.38	3.14	0.09	NS	NS	
c_*ε-Proteobacteria*	1.88	1.37	1.96	2.94	1.43	0.33	0.79	1.55	0	NS	NS	Only
c_*γ-Proteobacteria*	15.1	17.7	9.45	18.9	16.6	14.1	13.5	20.8	11.4	NS	<0.001	Enriched
f_*Succinivibrionaceae*^†^	7.12	9.76	5.30	9.02	8.37	6.43	6.58	16.0	11.3	NS	0.036	
g_*Ruminobacter*	2.24	2.38	1.11	2.03	3.18	2.24	1.51	2.19	0.19	NS	0.020	Enriched
o_*Pseudomonadales*	1.96	2.12	0.96	2.88	1.72	2.66	1.58	1.87	0.02	0.083	0.013	Enriched
f_*Moraxellaceae*	1.60	1.74	0.35	1.95	0.90	1.07	1.15	1.58	0.02	NS	0.051	Enriched
o_*Xanthomonadales*	4.36	3.52	2.26	5.55	3.66	3.11	3.81	1.65	0	NS	0.008	Only
f_*Sinobacteraceae*	4.25	2.56	1.92	4.77	3.10	2.97	3.64	1.53	0	NS	0.042	Only
g_*Nevskia*	2.74	1.56	1.36	2.29	1.41	1.55	1.49	1.06	0	0.067	NS	Only
*Spirochaetes*	2.90	1.73	5.69	2.80	1.12	3.02	1.89	2.35	6.45	0.035	0.005	
g_*Treponema*^†^	2.73	1.73	5.46	2.73	0.96	2.66	1.89	1.99	6.26	NS	NS	
*Synergistetes*	2.33	3.61	5.94	9.70	2.11	1.99	5.76	2.80	0.02	NS	NS	

* *Only the taxa over a relative abundance greater than 1% were shown*.

** *c, Class; o, Order; f, Family; g, Genus*.

# *Found only in the CAP. Those taxa exclusively found in the CAP of the in vitro monocultures of Ent. caudatum and Epi. caudatum are underlined*.

† *For those taxa, only the lowest classifiable taxa were shown because their higher taxa had similar relative abundance as the taxa shown*.

#### Methanogens

Collectively, methanogens belonging to the genera *Methanobrevibacter* and *Methanimicrococcus* and the class of *Thermoplasmata* were identified from the FLP of rumen fluid and the CAP of the fresh rumen fluid (Table 3B). Among all the 72 analyzed single ciliate cells of fresh rumen fluid, only 32 had one or more methanogen taxa detected, with 25 of the 72 CAP samples having detectable *Methanobrevibacter*, 8 having *Thermoplasmata*, and 1 having only *Methanimicrococcus* that was not detected in the FLP (a few CAP samples had more than one of the methanogen taxa detected). No significant difference in the relative abundance of each of the detected methanogen taxa was found among the 8 ciliate genera, between the CAP and the FLP, or among the five cows.

The qPCR analysis revealed the presence of *Thermoplasmata* in all the CAP and the FLP samples and the presence of *Methanosphaera stadtmanae* in the CAP of 4 of the 8 ciliate genera and the FLP of all the rumen fluid samples (Table 6). The relative abundance of total methanogens was higher in the FLP than in the CAP of all the identified ciliate genera (*P* < 0.001) except *Epidinium* and *Isotricha*. The relative abundance of the other 3 methanogen taxa did not differ between the FLP and the CAP of any of the 8 ciliate genera.

**Table 6:** Relative abundance (%) of methanogenic taxa in both FLP and CAP of freshly isolated ruminal ciliate cells as quantified using qPCR^*^.

	**CAP of individual ruminal ciliate genera**									***P-*****value**	
**Taxa**	***Dasytricha***	***Diplodinium***	***Diploplastron***	***Entodinium***	***Epidinium***	***Isotricha***	***Ophryoscolex***	***Polyplastron***	**FLP**	**Genera**	**CAP vs. FLP**
Total methanogens	0.153^b^	0.164^b^	0.135^b^	0.160^b^	0.251^ab^	0.416^ab^	0.212^b^	0.166^b^	0.891^a^	0.359	<0.001
*Methanobrevibacter*	0.104	0.112	0.071	0.052	0.166	0.104	0.160	0.041	0.260	0.300	<0.001
*Thermoplasmata*	0.035	0.103	0.014	0.013	0.019	0.193	0.110	0.055	0.335	0.520	<0.001
*M. stadtmanae*^#^	0.022	<0.001	<0.001	0.100	0.054	<0.001	<0.001	0.067	0.018	0.229	0.666

* *Data are shown as means of relative abundance of individual taxa relative to total prokaryotes. n = 9 for CAP of each ciliate genus and n = 5 for FLP*.

# *Methanosphaera stadtmanae*.

*Different superscripts within a row indicate significant differences (*P* < 0.05)*.

## Discussion

Living in the rumen where prokaryotes exist constantly at a very high density, at least 10^10^ bacteria per g of rumen content (calculated from Yu and Morrison, 2004; Kim and Yu, 2012) ruminal ciliates have the opulence of bacteria and archaea to choose as preys or symbiotic partners. During the evolution of ruminants over millions of years (Mackie, 2002), ruminal ciliates and other members of the ruminal microbiota, especially the prokaryotes, formed intriguing predator-prey and symbiotic relationships. Using high-throughput sequencing, this study represents the first effort to investigate the potential selectivity of ruminal ciliates toward preys and symbiotic partners. Certain bacteria, but not methanogenic archaea, appeared to be associated with ciliate cells, either enriched or exclusively found in ruminal ciliate cells. Those bacteria enriched in the ruminal ciliate cells may represent the preferred preys, while those exclusively found in the ruminal ciliate cells are probably symbionts.

### Differential prokaryotic populations inside and outside of ruminal ciliates

The two ciliate monocultures had very different FLP, but rather similar CAP (Figure 1A). Because the two monocultures were fed the same feed but grown in different media, the difference in the FLP and similarity in the CAP between the two ciliate species may suggest selectivity for prokaryotes either as preys or as symbionts. The small difference in the CAP between the two ciliate species might suggest different selectivity. *Entodinium* has smaller cell size than *Epidinium* and prefers small-sized starch granules (Williams and Coleman, 1997). It is conceivable that *Entodinium* selects small prokaryotic cells as preys due to its small size. *Entodinium* also has greater bacterivorous activity than other rumen ciliates (Coleman G. S. and Sandford, 1979). It remains to be determined if and to what extent the above morphological and behavioral differences contribute to prey preference and selection. It is also interesting to note that the profiles of the CAP-specific bacteria were rather stable overtime after feeding even though both ciliate species were incubated as batch monocultures. This suggests that the bacteria associated with each ciliate species are relatively stable and our single cell procedure can generate rather reproducible CAP profiles.

As shown in the ciliate monocultures, the FLP of the rumen fluid had greater species richness and diversity than the CAP. Compared to the FLP of the monocultures of *Ent. caudatum* and *Epi. caudatum*, the FLP of the rumen fluid had a much greater species richness and diversity (Table 1) but a lower predominance of *Proteobacteria* (Figure 3). The predominance of *Proteobacteria*, particularly γ*-Proteobacteria*, in the FLP of the monocultures, which was fed a wheat-based ciliate feed, is consistent with the increased occurrence of γ*-Proteobacteria* in the rumen of cattle fed a high grain diet (Petri et al., 2013). Interestingly, the CAP of the fresh rumen fluid also had a similar relative abundance of *Proteobacteria* as the CAP of the *in vitro* monocultures of *Ent. caudatum* and *Epi. caudatum*, and α*-Proteobacteria* was also detected as the most predominant class of CAP-specific bacteria (on average, over 14% of the CAP-specific bacteria were assigned to this class among all isolates). Evidently, ruminal ciliates prefer some members of *Proteobacteria*, especially α*-Proteobacteria*, as preys or symbiotic partners even when living in the presence of different FLP. This is another line of evidence for selective predation and/or symbiosis of ruminal ciliates. The finding of *Proteobacteria* as predominant CAP of the ruminal ciliates is consistent with this group of bacteria being predominant inside free-living ciliates (Görtz and Brigge, 1998). However, our results contradict the report of Irbis and Ushida (2004), who detected *Firmicutes* as the dominant bacteria (87.2%) followed by *Bacteroidetes* (10.6%). Future research is needed to determine if *Proteobacteria* is the preferred prey or endosymbionts of ruminal ciliates.

### Bacteria enriched in ruminal ciliate-associated prokaryotes

Selective predation has been demonstrated in fresh-living freshwater zooplankton, some nonflagellates, and ciliates, and the selectivity was attributed to size selection of the preys (Greene, 1983; Kinner et al., 1998; Matz and Kjelleberg, 2005). By comparing the overall rumen microbiotas between faunated and defaunated ruminants (Ushida et al., 1991; Ozutsumi et al., 2006), selective predation was also suggested for ruminal ciliates. In the present study, we found that some bacteria were significantly “enriched” in the CAP though they were also found in the FLP. The enriched bacteria were assigned to a small number of taxa including *Actinobacteria, Butyrivibrio, Proteobacteria*, β*-Proteobacteria, Burkholderiales*, and *Moraxellaceae*. Interestingly, similar enriched bacteria were found in the CAP of both monocultures maintained in laboratory and the fresh rumen fluid, and this congruence between laboratory monocultures and fresh isolates shall not be regarded as chance. Little is known about the selective predation of ruminal ciliates, and several studies done in the 1980's and 1990's showed that *Butyrivibrio fibrisolvens* and *Selenomonas ruminantium* were probably preferred preys for various ruminal ciliates (Coleman, 1964, 1986; Coleman and Laurie, 1974, 1977; Coleman G. and Sandford, 1979; Coleman G. S. and Sandford, 1979). In a few later studies (Coleman G. S. and Sandford, 1979; De la Fuente et al., 2011), however, several species of the entodiniomorphs (ex: *Ent. caudatum, Diplodinium dentatum*, and *Metadinium medium*) showed no preference in engulfing the tested bacterial species. Additionally, *Butyrivibrio* and several taxa of *Proteobacteria* were differently enriched among the different genera of the ruminal ciliates, suggesting different selectivity or preference of ruminal bacteria as preys. Collectively, the present study provided new evidence to pointing toward selective predation of ruminal bacteria by ruminal ciliates. Further studies using different approaches, such as using labeled bacteria, can help confirm selected predation and elucidate the underlying mechanism.

### Bacteria exclusively found in ruminal ciliate-associated prokaryotes

A number of bacterial taxa were only found in the CAP of both the laboratory ciliate monocultures and the fresh rumen fluid. Most of these “CAP-specific” bacteria belong to *Proteobacteria* (further discussed below). Some of the CAP-specific bacteria, including those classified to *Acidobacteria, Actinobacteria*, and *Sediminibacterium*, are common soil bacteria, but they were considered allochthonous, not autochthonous, in the rumen (Qu and Yuan, 2008; Henderson et al., 2015). The high prevalence and predominance of these bacteria exclusively found in the CAP of both ciliate monocultures and the fresh rumen fluid identified in the present study cannot be simply explained as contamination because they were not detected in the FLP.

Free-living ciliates can acquire endosymbiotic prokaryotes by vertical transmission and direct recruitment. Vertical transmission of methanogenic endosymbionts from one generation to the next has been reported in freshwater ciliates, such as species of *Metopus, Brachonella, Caenomorpha*, and intestinal (not ruminal) ciliates, such as *Nyctotherus ovalis* of cockroach (van Hoek et al., 2000). The vertically transmitted endosymbionts are often undetectable in FLP fraction (van Hoek et al., 2000). Direct recruitment of endosymbionts has been shown in anaerobic heterotrichous ciliates *Nyctotherus* spp. (van Hoek et al., 1999, 2000), and the directly recruited endosymbionts can be found in both FLP and CAP fractions. Also, replacement of endosymbiotic methanogens was reported in the monoxenic culture of *Trimyema compressum*, a free-living anaerobic ciliate, with the initial endosymbiotic bacteria (mostly *Firmicutes*) and methanogens (*Methanobacteriaceae*) being successfully replaced with *Methanobacterium formicicum* as its new endosymbionts (Wagener et al., 1990). The above acquisition mechanisms of both bacterial and archaeal endosymbionts were determined from research on diverse free-living non-ruminal ciliates. In the rumen, prokaryotes exist at a greater abundance (10^10^–10^11^/g of rumen content) and population stability than in other environments, such as marine (10^6^ cells/ml) (Amaral-Zettler et al., 2010) and freshwater (10^5^-10^7^ cells/ml) (White et al., 1991). The persistent high density of prokaryotes together with the vigorous bacterivorous activity of ruminal ciliates (Coleman G. S. and Sandford, 1979; De la Fuente et al., 2011) would make multiple acquisitions of free-living prokaryotes as endosymbionts more plausible in the rumen than in other habitats. However, the significant differences between the CAP and the FLP of both the *in vitro* monocultures and the fresh ruminal samples analyzed in the present study suggest possible vertical transmission. Moreover, the similar CAP-specific taxa found from the *in vitro* monocultures of *Ent. caudatum* and *Epi. caudatum* and from the single ciliate cells of fresh rumen fluid also suggest vertical transmission of endosymbionts. Specifically, the CAP-specific bacteria, especially those of *Proteobacteria* including α- and β*-Proteobacteria*, which are minor ruminal bacteria, were ubiquitous in all the CAP samples. In addition to their omnipresence in the CAP, rare detection of CAP-specific bacteria in the FLP supports the argument of their vertical transmission as endosymbionts. Analysis using FISH has detected prokaryotic cells outside food vacuoles but underneath the pellicle of some ruminal ciliates including *Ent. caudatum* (Valle et al., 2015), and intracellular replicating prokaryotic cells were also observed through transmission electron microscopy (Park et al., 2017), lending support to the presence of true endosymbionts of ruminal ciliates.

α*-Proteobacteria* were detected at high predominance in the CAP-specific bacteria (13.4% of the CAP of *Ent. caudatum*, 7.40% of the CAP of *Epi. caudatum*, and 5.17% of the CAP among the fresh isolates). α*-Proteobacteria* can resist digestion inside ciliate cells (Gong et al., 2016), and the mechanisms have been investigated, mostly in free-living ciliates (Görtz, 2001). It was speculated that some α*-Proteobacteria* can express certain periplasmic proteins, which can interact with phagosomal membranes or provide protection from lysis by inactivating host lytic enzymes. Indeed, certain bacteria have inherent phospholipid metabolism and protein secretion systems that afford resistance to digestion by ciliates and intra-ciliate survival by translocating effector proteins (Hubber et al., 2004; Lutz et al., 2015). Additionally, among the α- and γ*-Proteobacteria* associated with protists, types 6 and 4 secretion systems are known to allow them to survive inside ciliate host cells (Christie and Cascales, 2005; Pukatzki et al., 2006). Furthermore, coding genes of three types of lysozyme inhibitors have been found in the completed genomes of some α-, β-, and γ*-Proteobacteria* (Callewaert et al., 2012), and these bacteria were predominant in the CAP of both *Ent. caudatum* and *Epi. caudatum*. Therefore, the high predominance of *Proteobacteria*, particularly α*-Proteobacteria*, among the CAP-specific bacteria could be attributable to their resistance to digestion and/or their ability to replicate inside the ruminal ciliate cells (Gong et al., 2016).

*Devosia* was among the minor taxa (< 0.5% of total prokaryotes) of the CAP-specific bacteria. This genus belongs to α*-Proteobacteria*, and it contains the second obligate endosymbiont ever isolated from ciliate *Euplotes magicirratus* (Vannini et al., 2003). However, *Euplotes magicirratus* has never been detected in the rumen, and thus the occurrence of *Devosia* exclusively in the CAP of the single ruminal ciliate cells was intriguing. In our previous study (Park et al., 2017) and several other studies (Coleman, 1962; Hino and Kametaka, 1977; Bonhomme et al., 1982a,b), it was demonstrated that *Ent. caudatum* could not be grown or maintained in axenic cultures. Presumably, the antibiotics used to remove the free-living bacteria also killed the intracellular prokaryotes that are essential for the survival of host ciliates, as in the case where *Euplotes* depends on *Devosia* for survival (Vannini et al., 2003). Further research is warranted to determine if the intracellular prokaryotes detected in our study, particularly the members of *Proteobacteria* including *Devosia*–related bacteria are essential endosymbionts for the survival of host ciliates. Endosymbionts, including obligate endosymbionts, such as *Polynucleobacter*, have been reported for several species of ciliates (Soldo, 1987; Görtz, 2001). No endosymbionts have ever been reported for ruminal ciliates. Given that *Ent. caudatum* requires live prokaryotes for its survival (Fondevila and Dehority, 2001; Park et al., 2017), and repeated efforts have all failed to establish lasting axenic culture of ruminal ciliates (Coleman, 1962; Hino and Kametaka, 1977; Bonhomme et al., 1982a,b), it is tempting to speculate that certain species of these CAP-specific bacteria, especially those assigned to α*-Proteobacteria*, such as *Ellin329, Bradyrhizobiaceae* and *Rhodospirillaceae*, might be the essential prokaryotes for ruminal ciliates, at least *Ent. caudatum*, to survive.

### Association of methanogens with ruminal ciliate cells

Ruminal ciliates and methanogens (almost exclusively hydrogenotrophic methanogens) form mutualistic relationship via interspecies hydrogen transfer. This relationship contributes to methane production by rumen microbiome and thus has been a research focus. In the present study, methanogens were found at low relative abundance (of total prokaryotes), and no methanogen was enriched or found exclusively in the CAP of laboratory ciliate monocultures or the fresh rumen fluid, suggesting that the ruminal ciliates probably have no preference for methanogens as preys or vertically transmitted methanogenic endosymbionts. The methanogens detected in the CAP might be preys engulfed by the host ciliates. A couple of studies showed that only 8–40% the *Entodinium* cells examined had intracellular methanogens detectable (Vogels et al., 1980). Although at a higher frequency, other entodiniomorphids genera also did not have persistent detectable intracellular methanogens (Lloyd et al., 1996; Váradyová et al., 2001). Based on the results of the present study and previous studies (Finlay et al., 1994; Lloyd et al., 1996), ruminal ciliates probably do not have true persistent symbiotic methanogens or preference of methanogens as prey.

Endosymbionts have been reported surrounding hydrogenosomes of several anaerobic ciliates (Finlay et al., 1993; Shinzato et al., 2007). Such proximity can benefit both the ciliate host and the endosymbiotic methanogens. Among the ruminal ciliates analyzed in the present study, species of *Ent. caudatum, Entodinium simplex* (probably other species of *Entodinium*), and *Diploplastron affine* (probably other species of *Diploplastron*) contain no hydrogenosome, while species of *Epidinium* and other genera do have hydrogenosomes (Yarlett et al., 1984; Ellis et al., 1994). Hypothetically, the hydrogenosomes of *Epi. caudatum* and other genera can attract hydrogenotrophic methanogens as endosymbionts to consume the hydrogen released from the hydrogenosomes. In one study, production of hydrogen and/or formate increased after feeding, and free-living methanogens were attracted to the cytoplasm of ruminal ciliates (Tokura et al., 1997; Ushida, 2010). However, the relative abundance of *Methanobrevibacter* spp. in the CAP of *Epi. caudatum* decreased 2 h after feeding. In the present study, no difference was found in the occurrence or predominance of methanogens between the ruminal ciliates that contain hydrogenosomes and those contain no hydrogenosomes. Taken together, it is difficult to conclude if the detected methanogens are symbionts or engulfed preys. More research will be needed to verify the dynamic localization of methanogens associated with ruminal ciliates, especially hydrogenosome-carrying ciliates including *Epi. caudatum* and species of *Isotricha* and *Dasytricha*.

## Conclusion

Technical limitations make it extremely difficult to identify true symbionts, to distinguish true endosymbionts from engulfed preys, or to determine the selectivity of symbionts and preys. Using both monocultures maintained in laboratory and fresh rumen fluid, we identified bacteria, not methanogens, enriched or exclusively found in extensively washed single cells of ruminal ciliates. These results suggest selective predation on bacteria by ruminal ciliates and the presence of symbionts, including possible vertical transmission of symbionts. Future studies can incorporate controlled starvation of ciliates and live-dead cells detection of CAP, such as treatment of washed ciliate cells with propidium monoazide, which penetrates into cells with damaged cell membrane and render their DNA unamplifiable by PCR (Nocker et al., 2007), to help distinguish symbionts from engulfed preys that have damaged cell membrane. Additionally, the CAP-specific bacteria can be specifically targeted for detection and localization using specific probes designed from the 16S rRNA sequences, for characterization using single-cell genomics, or for isolation using new media designed from genomic information. Such studies can help elucidate the biological interactions between ciliates and prokaryotes and their role in rumen functions.

## Author contributions

TP was responsible for the overall study including sample collection, data analysis and drafting the manuscript. ZY supervised the study and revised the paper.

### Conflict of interest statement

The authors declare that the research was conducted in the absence of any commercial or financial relationships that could be construed as a potential conflict of interest.

## Abbreviations

FLP: free-living prokaryotes
CAP: ciliate-associated prokaryotes
rRNA: ribosomal RNA
FISH: fluorescence *in situ* hybridization
OTU: operational taxonomic unit
PCoA: principal coordinates analysis
ANOSIM: analysis of similarity
qPCR: quantitative polymerase chain reaction.
