# Supplementary material for: Do Ruminal Ciliates Select Their Preys and Prokaryotic Symbionts?
Source: Front Microbiol. 2018 Jul 31;9:1710. doi: 10.3389/fmicb.2018.01710 (PMC6079354; doi:10.3389/fmicb.2018.01710)
Supplement: Supplementary file 1 [file Table_1.DOCX]

Supplementary Material

Do ruminal ciliates select their preys and prokaryotic symbionts?

Running title: Prey and symbiont selection by ruminal ciliates

Tansol Park, Zhongtang Yu*

Department of Animal Sciences, The Ohio State University, Columbus OH 43210

*** Correspondence:** Zhongtang Yu: [yu.226@osu.edu](mailto:yu.226@osu.edu)

**Supplementary Data**

**Figure S1.** Methanogen taxa in the CAP of monocultures of *Ent. caudatum* and *Epi. caudatum* that showed significant temporal changes in relative abundances after feeding.

**Figure S2.** Light microscopic images of ruminal ciliate single cells isolated from Jersey dairy cows. **A**, *Dasytricha*; **B**, *Diplodinium*; **C**, *Diploplastron*; **D**, *Entodinium*; **E**. *Epidinium*; **F**, *Isotricha*; **G**, *Ophryoscolex*; **H**, *Polyplastron*. The scale bars = 10 μm.

| **Table S1.** GenBank accession numbers of the 18S rRNA gene sequences generated in the taxonomic confirmation of the isolated single cells of ruminal ciliates. | | | |
| --- | --- | --- | --- |
|  | Ciliate species or genus | Accession no. | Host animal |
| Monocultures | *Entodinium caudatum* | KY111861 | Gerenuk |
|  | *Epidinium caudatum* | KY111865 - KY111866 | Jersey dairy cow |
| Fresh isolates | *Dasytricha* | KY111870 - KY111878 | Jersey dairy cow |
|  | *Diplodinium* | KY111879 - KY111883 | Jersey dairy cow |
|  | *Diploplastron* | KY111896 - KY111904 | Jersey dairy cow |
|  | *Entodinium* | KY111852 - KY111860 | Jersey dairy cow |
|  | *Epidinium* | KY111862 - KY111864 | Jersey dairy cow |
|  | *Isotricha* | KY111867 - KY111869 | Jersey dairy cow |
|  | *Ophryoscolex* | KY111884 - KY111886 | Jersey dairy cow |
|  | *Polyplastron* | KY111887 - KY111895 | Jersey dairy cow |

| **Table S2.** Primers used in PCR and qPCR amplification of methanogen 16S rRNA genes. | | | | | |
| --- | --- | --- | --- | --- | --- |
| Target | Primer | Sequence, 5' to 3' | Annealing T  (°C) | Product size  (bp) | References |
| Total Archaea | Met86f | GCTCAGTAACACGTGG | 55 | 849 | (Tymensen and McAllister, 2012) |
|  | Met915r | GTGCTCCCCCGCCAATTCCT |  |  |  |
| Total Archaea  (for qPCR) | NestmetF | AMGWTCCAGGCCCTACGG | 60 | 149 | (Tymensen and McAllister, 2012) |
|  | NestmetR | TGGCACCSGTCTTRCCC |  |  |  |
| *Methanobrevibacter* | NestMbbF | TGGGAATTGCTGGWGATACTRTT | 60 or 63 | 231 | (Tymensen and McAllister, 2012) |
|  | NestMbbR | GGAGCRGCTCAAAGCCA |  |  |  |
| *Thermoplasmata* | NestRCCF | TTCTGGGGTAGGGGTAAAATC | 60 | 149 | (Tymensen and McAllister, 2012) |
|  | NestRCCR | GTCTGCAGCGTTTACACCCT |  |  |  |
| *Methanosarcinaceae* | Msc380F | GAAACCGYGATAAGGGGA | 60 | 408 | (Yu et al., 2005) |
|  | Msc828R | TAGCGARCATCGTTTACG |  |  |  |
| *Methanosphaera stadtmanae* | StadF | CTTAACTATAAGAATTGCTGGAG | 60 | 150 | (Carberry et al., 2014) |
|  | StadR | TTCGTTACTCACCGTCAAGATC |  |  |  |

| **Table S3.** Generic composition^#^ of ruminal ciliates in the collected rumen fluid samples. | | | | | | |
| --- | --- | --- | --- | --- | --- | --- |
| Genus |  | Cows | | | | |
|  |  | A | B | C | D | E |
| Total | Counts* | 14.389±0.950 | 6.080±0.371 | 5.824±0.088 | 4.443±0.135 | 12.907±0.305 |
|  |  |  |  |  |  |  |
| *Dasytricha* | Counts | 0.099±0.019 | - | 0.091±0.005 | 0.032±0.009 | 0.176±0.018 |
|  | (%)** | 0.681±0.108 | - | 1.558±0.097 | 0.727±0.225 | 1.358±0.111 |
| *Diplodinium* | Counts | 0.141±0.028 | - | 0.096±0.018 | - | 0.128±0.024 |
|  | (%) | 0.987±0.206 | - | 1.659±0.343 | - | 0.994±0.193 |
| *Diploplastron* | Counts | 0.067±0.003 | 0.001±0.000 | 0.043±0.005 | - | 0.075±0.019 |
|  | (%) | 0.468±0.039 | 0.009±0.002 | 0.733±0.093 | - | 0.584±0.163 |
| *Entodinium* | Counts | 13.563±0.840 | 6.068±0.377 | 5.523±0.126 | 4.373±0.144 | 12.281±0.282 |
|  | (%) | 94.303±0.375 | 99.797±0.188 | 94.814±0.835 | 98.424±0.277 | 95.156±0.192 |
| *Epidinium* | Counts | 0.021±0.005 | - | 0.011±0.011 | - | 0.021±0.011 |
|  | (%) | 0.145±0.026 | - | 0.188±0.188 | - | 0.165±0.083 |
| *Isotricha* | Counts | 0.091±0.014 | - | 0.021±0.005 | 0.037±0.014 | 0.091±0.030 |
|  | (%) | 0.624±0.069 | - | 0.364±0.086 | 0.849±0.323 | 0.702±0.227 |
| *Ophryoscolex* | Counts | 0.032±0.005 | - | 0.011±0.005 | - | 0.016±0.009 |
|  | (%) | 0.228±0.045 | - | 0.181±0.090 | - | 0.122±0.069 |
| *Polyplastron* | Counts | 0.003±0.003 | 0.011±0.011 | 0.002±0.001 | - | 0.002±0 |
|  | (%) | 0.016±0.016 | 0.194±0.187 | 0.037±0.010 | - | 0.012±0 |
| Others | Counts | 0.373±0.087 | - | 0.027±0.019 | - | 0.117±0.019 |
|  | (%) | 2.547±0.433 | - | 0.466±0.341 | - | 0.905±0.138 |
| ^#^ shown as mean ± standard error of the mean.  * counts ×10^5^ cells/ml of rumen fluid.  ** % of total ruminal ciliates. | | | | | | |

**References**

Carberry, C.A., Kenny, D.A., Kelly, A.K., and Waters, S.M. (2014). Quantitative analysis of ruminal methanogenic microbial populations in beef cattle divergent in phenotypic residual feed intake (RFI) offered contrasting diets. *Journal of Animal Science and Biotechnology* 5**,** 41.

Tymensen, L.D., and Mcallister, T.A. (2012). Community structure analysis of methanogens associated with rumen protozoa reveals bias in universal archaeal primers. *Appl Environ Microbiol* 78**,** 4051-4056.

Yu, Y., Lee, C., Kim, J., and Hwang, S. (2005). Group‐specific primer and probe sets to detect methanogenic communities using quantitative real‐time polymerase chain reaction. *Biotechnology and bioengineering* 89**,** 670-679.
