# Supplementary material for: Do Ruminal Ciliates Select Their Preys and Prokaryotic Symbionts?
Source: Front Microbiol. 2018 Jul 31;9:1710. doi: 10.3389/fmicb.2018.01710 (PMC6079354; doi:10.3389/fmicb.2018.01710)
Supplement: Supplementary file 2 [file Data_Sheet_1.pdf]

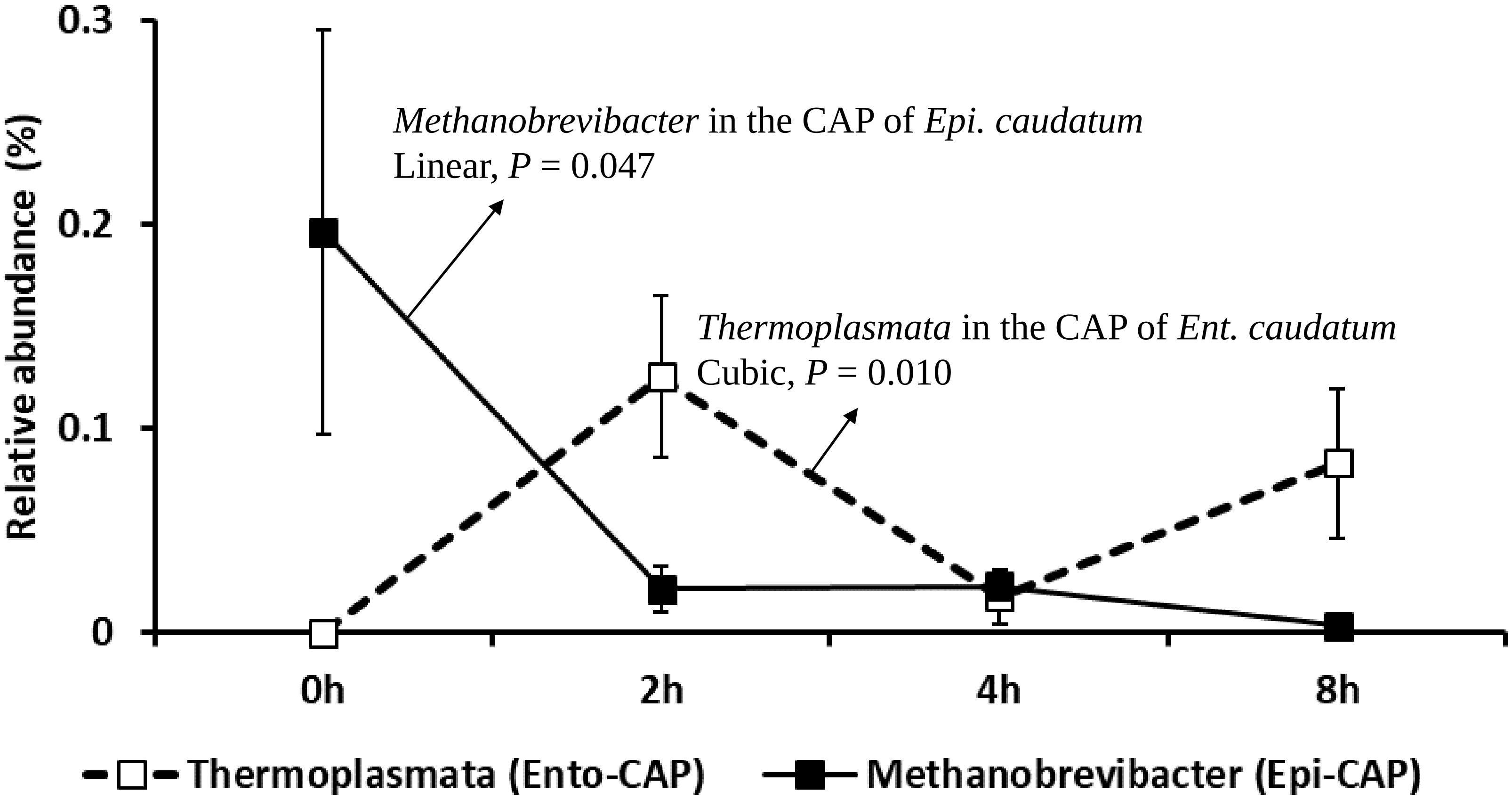

Figure S1. Methanogens taxa in the CAP of monocultures of *Ent. caudatum* and *Epi. caudatum* that showed significant temporal changes in relative abundances after feeding.
