# Supplementary material for: Do Ruminal Ciliates Select Their Preys and Prokaryotic Symbionts?
Source: Front Microbiol. 2018 Jul 31;9:1710. doi: 10.3389/fmicb.2018.01710 (PMC6079354; doi:10.3389/fmicb.2018.01710)
Supplement: Supplementary file 3 [file Data_Sheet_2.PDF]

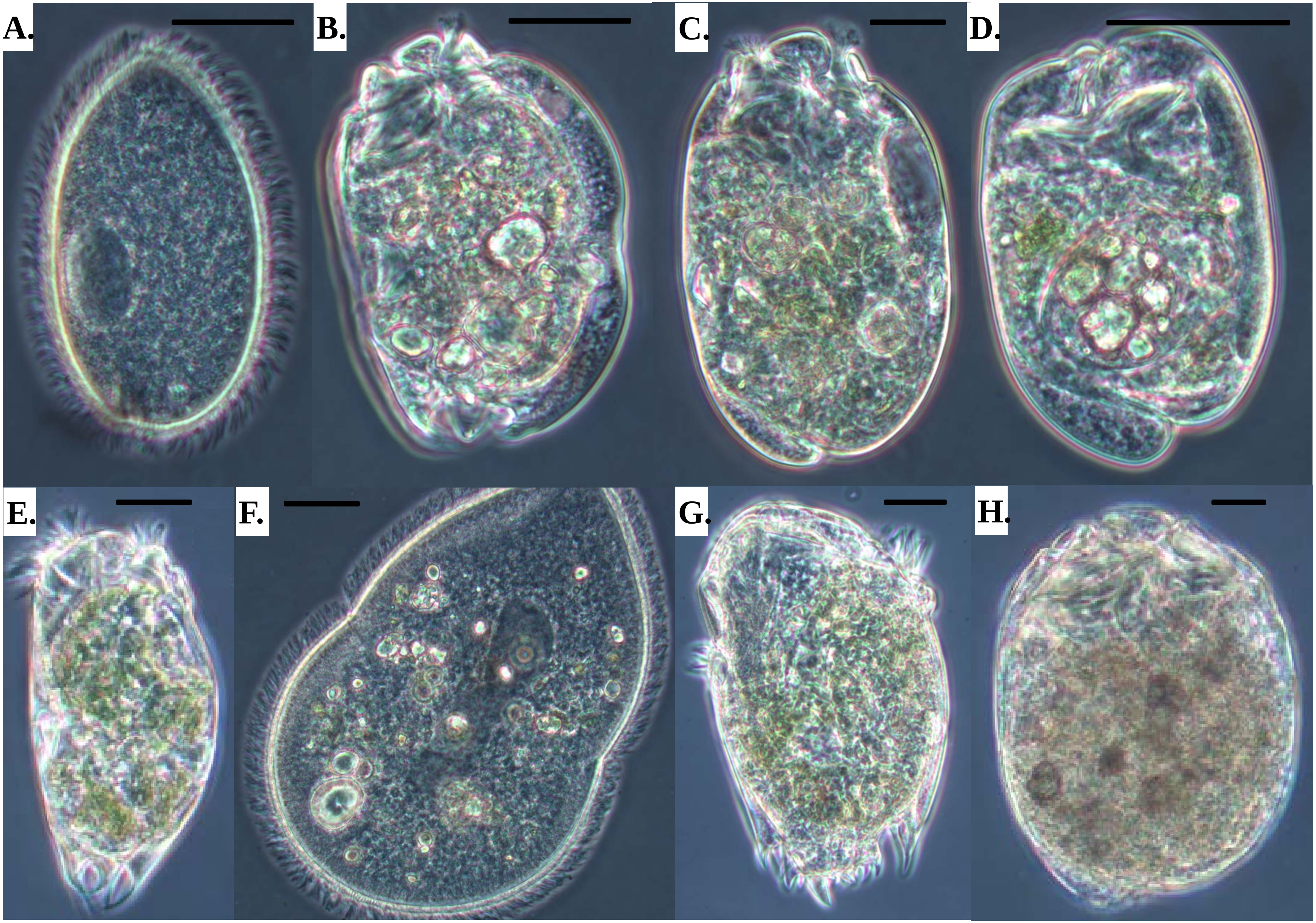

Figure S2. Light microscopic images of ruminal ciliate single cells isolated from Jersey dairy cows. **A**, *Dasytricha*; **B**, *Diplodinium*; **C**, *Diploplastron*; **D**, *Entodinium*; **E**. *Epidinium*; **F**, *Isotricha*; **G**, *Ophryoscolex*; **H**, *Polyplastron*. The scale bars = 10 μm.
